# Elevated cobalt levels in metal-on-polyethylene knee megaprostheses: a prospective 1-year cohort study of 56 patients with hip and knee megaprostheses

**DOI:** 10.2340/17453674.2024.40502

**Published:** 2024-04-17

**Authors:** Sarah Stammose FREUND, Andrea Pohly Jeppesen THORN, Ajay PURI, Michael Mørk PETERSEN, Thomas BAAD-HANSEN

**Affiliations:** 1 Department of Orthopedic Oncology, Aarhus University Hospital, Denmark; 2 Musculoskeletal Tumor Section of the Department of Orthopedic Surgery, Rigshospitalet – University of Copenhagen, Denmark; 3 Orthopedic Oncology, Tata Memorial Centre, HBNI, Mumbai, India

## Abstract

**Background and purpose:**

Concerns have emerged regarding elevated levels of cobalt and chromium in patients with metal-on-metal megaprostheses. This prospective study aims to identify systemic cobalt and chromium levels in metal-on-polyethylene knee and hip megaprostheses and their associations with other factors.

**Methods:**

56 patients underwent knee or hip megaprosthesis surgery at 2 sarcoma centers. Serum cobalt and chromium levels were measured preoperatively and thrice within the first year using inductively coupled plasma mass spectrometry.

**Results:**

A statistically significant difference in serum cobalt levels (1.4 ppb; 95% confidence interval [CI] 0.0–3.3) was observed 1 year after knee megaprosthesis surgery compared with preoperative levels. In contrast no difference in chromium levels was observed after 1 year compared with preoperative levels (0.05 ppb; CI 0.0–0.8). An association between younger age, higher eGFR, and increased cobalt levels was observed. No significant correlations were found between ion levels and resection length or the number of modular connections.

**Conclusion:**

We found elevated serum ion levels in metal-on-polyethylene knee megaprostheses in contrast to metal-on-polyethylene hip megaprostheses. Furthermore, a positive correlation between cobalt and chromium levels, and between cobalt and eGFR was identified, along with a negative correlation between cobalt and age. This study highlights the importance of monitoring systemic cobalt and chromium levels in patients with megaprostheses.

Megaprostheses primarily serve in the treatment of patients with malignant bone tumors but also find application in managing extensive bone defects, such as in revision joint replacement surgery and trauma surgery. Local and systemic toxic levels of cobalt (Co) and chromium (Cr) have been of special concern since the reports of adverse reactions to metal debris (ARMDs) in patients with metal-on-metal (MoM) total hip arthroplasty (THA) [1-3]. In 2012, a medical device alert [4] and an international consensus statement [5] were released in response to increasing evidence of risks associated with MoM implants. Consequently, joint replacement with MoM prostheses has been significantly reduced; however ARMDs due to trunnionosis at the taper connection are still reported in up to 4.7% in revision surgery [6].

Elevated levels of Co and Cr have been associated with chromosomal aberrations, delayed-type hypersensitivity responses, nephrotoxic effects, and potential teratogenic impacts [7-9]. A few case reports have documented both multiple organ failure and death resulting from Co toxicity induced by MoM THA [10,11]. An international threshold for clinical concern in patients with MoM THA has been established at between 2 and 7 parts per billion (ppb) in whole blood. Close observation is recommended within this range [4,5], though significant systemic adverse effects have only been observed in blood Co concentrations above 300 ppb [12].

A few studies have investigated the systemic concentrations of Co and Cr in relation to MoM megaprostheses. Laitinen et al. [13] observed significantly increased systemic metal ion levels, along with an elevated risk of developing ARMDs in patients treated with MoM knee megaprostheses. Furthermore, Friesenbichler et al. [14] demonstrated increased serum ion levels in patients with knee megaprostheses compared with conventional rotating-hinge knee arthroplasty. These studies raise concerns regarding the utilization of megaprosthesis due to their release of Co and Cr.

The objective of this study is to investigate the systemic concentrations of Co and Cr in various metal-on-polyethylene (MoP) megaprostheses used in hip and knee reconstruction at specified time intervals following the surgical procedures. Potential associations between Co/Cr levels and age, sex, body mass index (BMI), estimated glomerular filtration rate (eGFR), resection length, and the number of modular connections were investigated.

## Methods

### Design and study population

This prospective cohort study was conducted from April 2019 to August 2023. All patients scheduled for hip or knee megaprosthesis surgery at 2 sarcoma centers were assessed for eligibility (one center started inclusion from December 2022). Exclusion criteria included remaining life expectancy < 12 months, prior prosthetic surgery, or eGFR < 30 mL/min/1.73 m^2^ [15]. All patients completed a metal exposure questionnaire to rule out other potential sources as the cause of elevated metal ion values.

The procedures and megaprostheses utilized included:

- THA with Modular Universal Tumor and Revision System (MUTARS, Implantcast GmbH, Buxtehude, Germany) (THA-MUTARS);
- THA with Zimmer Segmental System (Zimmer, Warsaw, IN, USA) (THA-ZSS);
- Hemi hip arthroplasty with MUTARS (HEMI-MUTARS);
- Total knee arthroplasty (TKA) with Global Modular Replacement System (GMRS, Stryker, Kalamazoo, MI, USA) (TKA-GMRS);
- TKA with Zimmer Segmental System (TKA-ZSS).

The articulating components were manufactured using a CoCr alloy in combination with ultra-high-molecular-weight polyethylene (UHMWPE) at the joints. Tapers/modular connections of all prostheses were manufactured using a CoCr alloy, along with the stems in THA-ZSS and TKA-ZSS. Modular segments, stems in THA-MUTARS, HEMI-MUTARS, TKA-GMRS, and other connecting components were fabricated from Ti-6Al-4V alloy.

### Blood collection and analysis

Blood samples were collected preoperatively and at 4-, 8-, and 12-month follow-up. Stainless-steel needles were used for blood collection. The first 2 mL were discarded, and the samples were collected in no-additive BD Vacutainer tubes for trace element determination. All samples underwent centrifugation at 2,000 g on the same day and were transferred into separate 6 mL Vacutainer trace metal tubes. The samples were either refrigerated for a maximum of 7 days or stored at –18°C until analysis.

Co and Cr levels in serum were measured using inductively coupled plasma mass spectrometry (ICP-MS) at 2 different laboratories.

The levels of metal ions in serum were recorded in concentrations expressed as micrograms per liter (µg/L) or nanomoles per liter (nmol/L). To standardize the units of measurement, nmol/L was converted to ppb using the formula:

ppb = (nmol/L) * (molar mass of Cr or Co / 1,000)

Due to the reduced sensitivity of the measurement method at low serum ion values, measured values below 10 nmol/L and 1 µg/L are recorded as 0.5 ppb.

### Statistics

Histograms, probability plots, and Shapiro–Wilk tests were applied to assess data distribution. In case of serious deviations from a Gaussian distribution, various transformations were applied in an attempt to achieve an acceptable fit to a Gaussian distribution. A significant portion of the Co and Cr measurements share identical values, posing challenges in achieving a satisfactory fit to a Gaussian distribution irrespective of the applied transformation. Normally distributed data is described using means and standard deviations (SD) and non-normally distributed data is described using medians and ranges. Normally distributed outcomes were analyzed by a mixed model for repeated measurements with time as a fixed effect and patient as a random effect. Tukey’s multiple comparisons test was applied if a difference between outcomes was observed. For non-normally distributed outcomes that could not be successfully transformed, the Skillings–Mack test was applied. If the test indicated a difference between groups, the Wilcoxon matched-pairs signed-rank test was applied.

Model diagnostics confirmed linearity in residual plots and validated normality through Q–Q plots and histograms, affirming the robustness of our repeated measures mixed-effects model. Spearman’s rank correlation was used to determine correlations between variables.

Patient inclusion was based on a power calculation conducted for analyses within the broader context of an ongoing, yet unpublished, PhD initiative.

Analyses were performed using Stata version 17.0 (StatCorp, College Station, TX, USA) and GraphPad Prism version 10.1.1 (https://www.graphpad.com/features). This study was reported according to STROBE guidelines.

### Missing data

**Figure 1:**
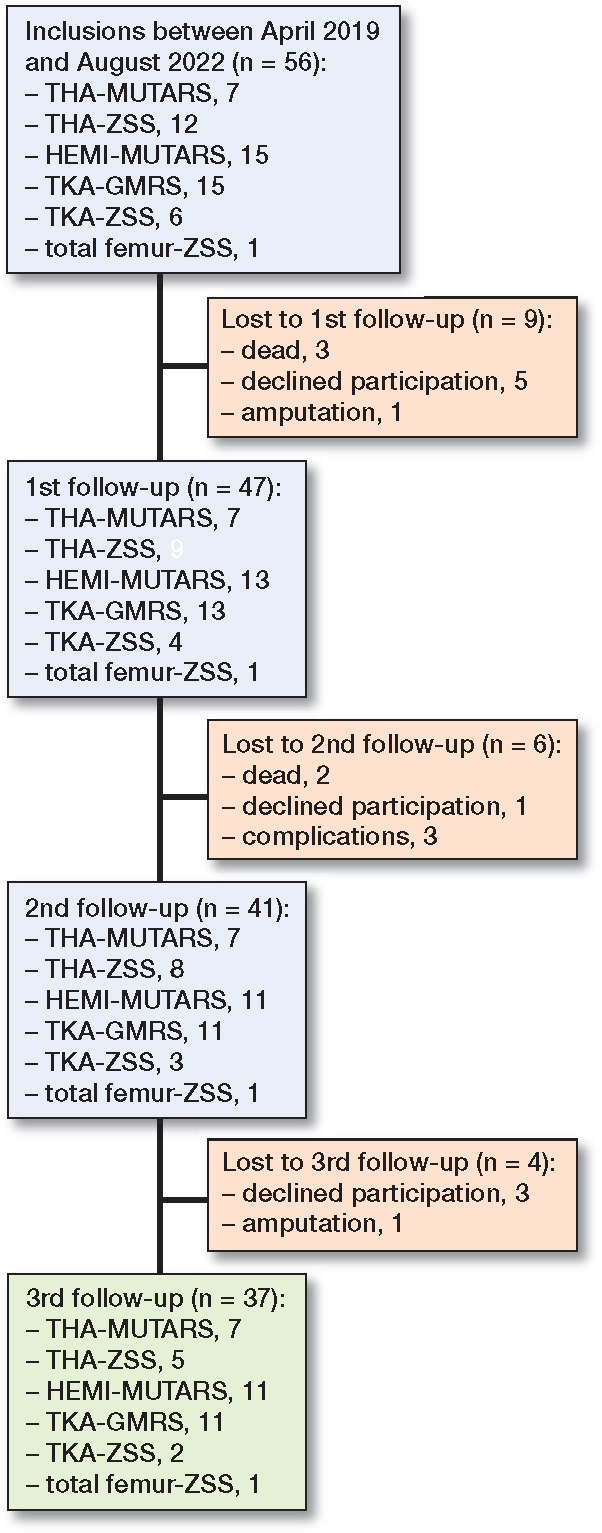
Inclusion flowchart.

In our analysis based on an initial intention-to-infer population of N = 56 (Figure 1), we addressed missing non-normally distributed data by omitting single observations with missing values. Importantly, we assumed that these missing values occurred at random. We refrained from using listwise deletion or imputation techniques, ensuring a straightforward representation of the available data. Furthermore, using mixed-model analysis on normally distributed data is considered comparable in validity to the multiple imputation approach [16].

### Ethics, data sharing, funding, and disclosures

The study is in accordance with the Helsinki Declaration and has been approved by the local ethics committee (No. 1-10-72-268-18) as well as the Danish Data Protection Agency (No. 1-16-02-363-18). All patients provided written consent prior to inclusion. Anonymized data can be obtained on request. The study received support from an unrestricted grant by Fisher Medical and Holms Mindelegat. The authors have no conflicts of interest to declare. Complete disclosure of interest forms according to ICMJE are available on the article page, doi: 10.2340/17453674.2024.40502

## Results

**Figure 2:**
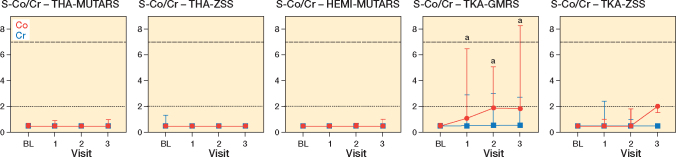
Median with range of serum cobalt (red circle) and chromium (blue square) levels in parts per billion over the first year in patients treated with various types of lower extremity megaprostheses. ^a^ Significant increase compared with baseline. The dotted and dashed horizontal lines represent the lower and upper thresholds for cobalt, indicating clinical concerns [4,5].

**Figure 3:**
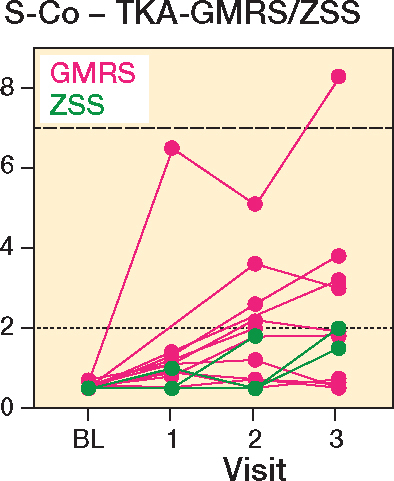
Individual serum cobalt levels (ppb) of GMRS and ZSS knee megaprostheses. For dotted and dashed horizontal lines, see Figure 2.

56 patients (34 men, 22 women) were included in the study, with 7 receiving a THA-MUTARS, 12 receiving a THA-ZSS, 15 receiving a HEMI-MUTARS, 15 receiving a TKA-GMRS, 6 receiving a TKA-ZSS, and 1 receiving a total femur-ZSS (Figure 1). A total of 19 patients were lost to follow-up: 9 at the first follow-up (3 dead, 5 declined participation, 1 amputation), 6 at the second follow-up (2 dead, 1 declined participation, 3 complications), and 4 at the third follow-up (3 declined participation, 1 amputation). Mean age at the time of surgery was 62 years (SD 16) and mean resection length was 15.2 cm (SD 5.7) (Table 1). In TKA-GMRS, the median Co levels showed an increase over time with a median difference of 0.4 ppb, CI 0.3–0.9; 1.4 ppb, CI 0.1–3.1; and 1.4 ppb, CI 0.0–3.3 at visits 1, 2, and 3, respectively (Figure 2). The median Co levels in TKA-ZSS also showed an increase at visit 3, with a median difference of 1.5 ppb compared with baseline; however, this difference was not statistically significant (Figure 2). None of the implants exhibited median Co levels above 2 ppb. Nevertheless, at visits 2 and 3, 4 patients with TKA-GMRS had serum Co levels above 2 ppb, with 1 patient exceeding 7 ppb at visit 3 (Figure 3).

**Table 1:** Patient demographic data gathered at the baseline assessment. Values are mean (standard deviation) or count

	Total (n = 56)	THA-MUTARS (n = 7)	THA-ZSS (n = 12)	HEMI-MUTARS (n = 15)	TKA-GMRS (n = 15)	TKA-ZSS (n = 6)	Total femur-ZSS (n = 1)
Sex, n (male:female)	34:22	7:0	10:2	6:9	8:7	2:4	1:0
Age	62 (16)	71 (7)	70 (10)	66 (11)	51(17)	53 (27)	69
BMI	27.5 (4.9)	29.0 (4.2)	26.2 (3.2)	26.3 (5.0)	29.3 (6.0)	27.9 (5.5)	22.2
Side, n (right:left)	26:30	3:4	6:6	7:8	5:10	4:2	1:0
Follow-up, days							
Baseline	–2 (6)	–6 (14)	–1 (2)	–1 (5)	1 (2)	–4 (3)	–2
Visit 1	124 (15)	119 (10)	134 (13)	122 (17)	123 (16)	122 (7)	133
Visit 2	246 (29)	258 (44)	240 (48)	247 (22)	243 (17)	245 (4)	258
Visit 3	396 (62)	444 (110)	392 (35)	379 (25)	392 (40)	345 (98)	383
Resection length (cm)	15.2 (5.7)	16.8 (4.7)	15.8 (4.0)	13.7 (2.5)	13.1 (2.9)	15.8 (3.7)	48
Location, n							
Proximal femur	34	7	12	15	0	0	1
Distal femur	14	0	0	0	9	5	0
Proximal tibia	7	0	0	0	6	1	0
Total femur	1	0	0	0	0	0	0
Indication for surgery, n							
Malignant tumor	14	0	2	0	8	3	1
Benign tumor	3	0	0	0	2	1	0
Metastases	32	3	10	14	3	0	0
Other	7	4	0	1	2	2	0
Modular connections, n							
1	8	2	0	3	3	0	0
2	36	3	7	12	11	3	0
3	5	2	0	0	1	2	0
4	7	0	5	0	0	1	1

Median Cr levels in THA-MUTARS, THA-ZSS, HEMI-MUTARS, TKA-GMRS, and TKA-ZSS displayed no change over time. A similar trend was observed regarding Co for THA-MUTARS, THA-ZSS, and HEMI-MUTARS (Figure 2).

**Figure 4:**
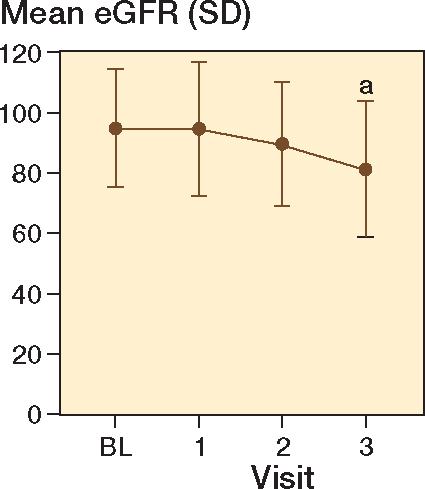
Mean estimated glomerular filtration rate (eGFR) over time with whiskers indicating standard deviation (SD). ^a^ Significant difference compared with baseline (mixed-effect model analysis).

Mixed-effect model analysis of eGFR repeated measures displayed a decrease in eGFR with a mean difference of 13 mL/min/1.73 m^2^ (CI 7–20) when comparing visit 3 with baseline (Figure 4). Mean eGFR is displayed in Table 2.

**Table 2:** Mean estimated glomerular filtration rate (mL/min/1.73 m^2^, eGFR) with (standard deviation) over time in various types of lower extremity megaprostheses

	Total (n = 56)	THA-MUTARS (n = 7)	THA-ZSS (n = 12)	HEMI-MUTARS (n = 15)	TKA-GMRS (n = 15)	TKA-ZSS (n = 6)	Total femur-ZSS (n = 1)
Baseline	95 (20)	81 (18)	90 (15)	89 (16)	111 (16)	103 (25)	87
Visit 1	95 (22)	78 (24)	84 (19)	89 (15)	114 (11)	116 (32)	–
Visit 2	90 (20)	89 (16)	67 (9)	86 (20)	106 (14)	96 (0)	–
Visit 3	81 (22)	74 (26)	64 (13)	79 (21)	94 (22)	82 (0)	87

**Figure 5:**
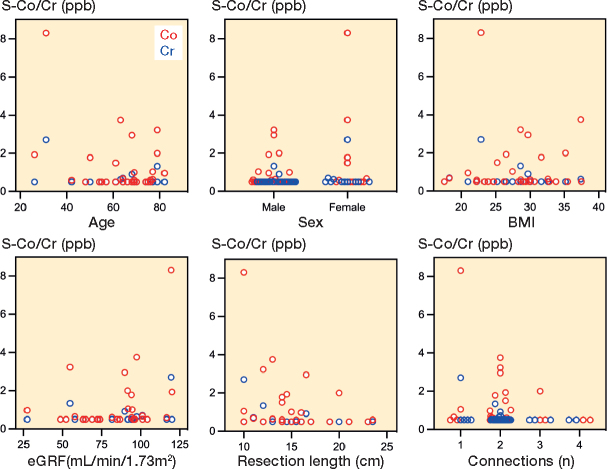
Scatter plots showing associations between serum cobalt (red)/ chromium (blue) levels and demographics/procedural variables at visit 3: age (Co: r –0.2, P = 0.01, Cr: r –0.2, P = 0.05), sex (Co: r 0.1, P = 0.2, Cr: r 0.07, P = 0.4), BMI (Co: r 0.1, P = 0.1, Cr: r –0.1, P = 0.2), eGFR (Co: r 0.3, P = 0.003, Cr: r 0.1, P = 0.1), resection length (Co: r –0.2, P = 0.6, Cr: r –0.1, P = 0.1), and the number of modular connections (Co: r –0.2, P = 0.08, Cr: r –0.1, P = 0.3).

A positive correlation was observed between Co and Cr levels (r = 0.4, P < 0.001), as well as between Co and eGFR (r = 0.3, P = 0.003). A negative correlation was noted between Co and age (r = –0.2, P = 0.01). However, no correlations were found between Cr and eGFR or Cr and age (eGFR: P = 0.1, age: P = 0.053). Additionally, there were no correlations between Co/Cr levels and the remaining demographic factors (sex: P = 0.2 and 0.4, BMI: P = 0.1 and 0.2), or with procedural factors such as resection length (P = 0.06 and 0.1) and number of modular connections (P = 0.08 and 0.3) (Figure 5).

## Discussion

In this prospective cohort study, we assessed changes in Co and Cr levels over time, involving various prosthetic systems, including THA-MUTARS, THA-ZSS, HEMI-MUTARS, TKA-GMRS, and TKA-ZSS. The research also investigated possible connections and relationships with demographic and procedural variables. We observed a median increase in Co levels over time in TKA-GMRS and TKA-ZSS, when compared with preoperative levels. 4 patients in the TKA-GMRS group had Co levels above 2 ppb at visits 2 and 3, one of which was above 7 ppb. There was a positive correlation between Co and Cr levels, and Co and eGFR. Additionally, a negative correlation was observed between Co and age. No correlations were detected between ion levels and other variables, specifically resection length and the number of connections.

Extensive research has been conducted regarding MoM THA; however, only a limited number of studies have examined the systemic levels of Co and Cr following surgery involving megaprostheses. Laitinen et al. [13] compared whole-blood levels of Co and Cr in patients treated with MUTARS MoM TKA and MUTARS MoP TKA. In line with our study, they reported a mean Co of 1.9 ppb, with the upper range reaching 7.3 ppb after 1 year in the MoP TKA group. Friesenbichler et al. [14] reported significantly elevated serum levels of Co and Cr in knee MoM megaprostheses compared with patients who underwent conventional rotating-hinge knee arthroplasty. The authors suggested that the elevation in ion levels could be attributed in part to friction at the modular connections, abrasive wear of soft tissue, and corrosion of the extensive non-articulating surfaces, as described by Jakobsen et al., where metal ion release was observed after implantation of CoCrMo alloy implants intramuscularly and intraabdominally in rats [17]. However, a later pediatric study by the same group [18] did not reveal any correlation with resection length, which corresponds with our findings. Although Laitinen et al. did not perform a correlation analysis regarding Co/Cr and prosthesis size, they suggested that the elevated serum ion levels in the study by Friesenbichler et al. were mainly due to the MoM TKA hinge mechanism. This was attributed to the fact that the only components constructed from a CoCrMo alloy were the articulating parts. None of the studies incorporated preoperative serum ion levels. Furthermore, the analyses were conducted using different methods (whole blood and serum). Consequently, comparing the results is not feasible, as ion metal levels are typically higher in plasma than in whole blood, and the measurements from these 2 sources are not interchangeable or readily convertible [19,20].

A recent cross-sectional study by Houdek et al. [21] investigated the serum Co and Cr levels of different MoP megaprostheses. 17 patients (63%) showed elevated serum ion levels above 1 ppb, of which 13 had been treated with TKA GMRS. 3 patients had Co levels above 7 ppm, which is the internationally accepted threshold of both Co and Cr [4,22,23]. These findings support our results, showing an increase in systemic Co levels in MoP TKA. Considering that the CoCrMo alloy was used in the articulating components, tapers, and stems of the ZSS systems, and no correlation between the increase in Co/Cr levels and other variables could be proven (see Figure 5)—especially resection length and the number of modular connections (tapers)—we posit that the elevation in Co/Cr levels is primarily linked to mechanical wear stemming from the articulating coupling mechanism, rather than being predominantly caused by taper fretting or corrosion of the surfaces.

Systemic accumulation of Co and Cr due to decreased kidney function has been suggested [24]. However, a recent study found a positive correlation between Co/Cr and eGFR, suggesting an increase in ion levels in patients with better kidney function [15]. Despite the observed decrease in mean eGFR in this study, a positive correlation between Co and eGFR was proven along with a negative correlation between Co and age. This could be explained by increased activity in younger patients with better kidney function, as seen in previous studies [25,26].

### Limitations

This study is limited by the small study population and the utilization of multiple prosthesis types. Given the characteristics of the patient cohort, including malignant disease and COVID-19, statistical adjustments were necessary to address the missing data. We assessed systemic ion levels at 4, 8, and 12 months following surgery. However, the mean time since surgery varies due to unexpected changes in the follow-up dates. Previous research has demonstrated an initial increase in systemic Co and Cr ion levels during the first year after MoM THA, followed by a subsequent decline observed between 1 and 6 years post-implantation [27,28]. Nonetheless, elevated levels have been noted several years after the surgery [13,14,21,29]. This raises the question of whether our study would have observed further increases in Co/Cr levels with an extended follow-up period.

Because we report data in serum, it is important to note that our results cannot be directly compared with similar studies conducted using whole blood.

### Conclusion

We observed a significant rise in serum ion levels in patients with MoP knee megaprosthesis during the first year after surgery. Younger age and higher eGFR were associated with increased ion levels, suggesting that patients with higher activity levels may contribute to metal release. No correlation between Co/Cr and the resection length or the number of modular connections could be proven, diminishing the likelihood of surface corrosion and fretting as the underlying causes for the increased serum ion levels.

In perspective, prolonged follow-up of these megaprostheses is essential to assess the long-term consequences of heightened systemic Co/Cr exposure.

Currently, there are no existing guidelines for managing elevated systemic Co and Cr levels in MoP megaprostheses. We advocate for the establishment of a consensus within the international orthopedic oncology community regarding the management of Co/Cr levels in patients undergoing MoP megaprosthesis treatment.

## Notes

Contributions are consistent with the 4 criteria of ICMJE. Design of study: SSF, TBH. Data acquisition: SSF, APT. Data analysis: SSF. Data interpretation: SSF, APT, AP, MMP, TBH. Paper draft: SSF. Critical review: APT, AP, MMP, TBH.

The authors would like to thank statistician Therese Koops Grønborg, Department of Clinical Medicine, Aarhus University for statistical advice.

Handling co-editors: Keijo Mäkelä and Robin Christensen

*Acta* thanks Minna K Laitinen and Jyrki Juhani Nieminen for help with peer review of this manuscript.
